# Supplementary material for: Life-History and Ecological Correlates of Egg and Clutch Mass Variation in Sympatric Bird Species at High Altitude
Source: Biology (Basel). 2023 Oct 2;12(10):1303. doi: 10.3390/biology12101303 (PMC10604263; doi:10.3390/biology12101303)
Supplement: Supplementary file 1 [file biology-12-01303-s001.zip › biology-2611014-supplementary.pdf]

Table S1. Life history traits and ecological factors of 22 bird species in sympatric area at high altitude

Species	Clutch size	Egg mass	Body mass	Development time	Predation rate	Life span#	*No.* of nests	Data quality	References
Horned Lark *Eremophila alpestris*	2.52	3.19	33.2	22.3	0.685	8	73	3	Li et al. 2016a,
Oriental Skylark *Alauda gulgula*	3.30	3.27	31.5	22.1	0.587	10.1	46	3	Li et al. 2015a<br>Li et al. 2018b
Citrine Wagtail *Motacilla citreola*	4.30	2.25	22.3	23.5	0.727	10.2	11	2	Current study
Tibetan Ground-Jay *Pseudopodoces humilis*	6.80	3.11	38.1	39.1	0.09	8	187	3	Li et al. 2015b<br>Liu et al. 2021
Black Redstart *Phoenicurus ochruros*	4.80	2.22	18.7	30.6	0.259	10.2	27	2	Current study
Eurasian Tree Sparrow *Passer montanus*	4.05	2.25	21.2	27.4	0.289	13.1	38	3	Li et al. 2022
White-rumped Snowfinch *Montifringilla taczanowskii*	4.60	3.56	38.2	34.6	0.167	8	6	1	Current study
Rufous-necked Snowfinch *Montifringilla ruficollis*	4.20	3.21	29.1	34.5	0.375	7	8	1	Current study
Twite *Carduelis flavirostris*	4.50	1.56	12.9	26.6	0.513	6.1	47	3	Current study
Streaked Rosefinch *Carpodacus rubicilloides*	3.50	3.30	41.7	28.8	0.538	12.8	13	2	Current study
Isabelline Wheatear *Oenanthe isabellina*	5.10	3.15	28.1	32.0	0.19	10.1	31	3	Li and Lu 2012b<br>Li et al. 2018a

Small Snowfinch *Montifringilla davidiana*	5.80	2.28	20.9	31.6	0.14	6	29	3	Li et al. 2013
Sand Martin *Riparia riparia*	4.90	1.40	13.8	38.0	0.105	10.1	19	2	Li et al. 2016b
Mongolian Lark *Melanocorypha mongolica*	3.30	4.30	58.3	23.0	0.5	25	4	1	Current study
Rock Sparrow *Petronia petronia*	5.10	3.05	29.8	32.6	0.11	7	35	3	Li and Lu 2012a
Brown Accentor *Prunella fulvescens*	3.40	2.13	19.5	26.8	0.541	14.3	37	3	Huang *et al*. 2020
Little Owl *Athene noctua*	3.00	17.67	177.5	52.0	0	15.6	4	1	Current study
Common Kestrel *Falco tinnunculus*	4.70	23.94	194.5	59.5	0	23.8	3	1	Current study
Eurasian Hoopoe *Upupa epops*	5.30	4.90	63.8	45.0	0.5	11.1	4	1	Current study
Rufous-breasted Accentor *Prunella strophiata*	3.25	1.93	18.4	26.4	0.692	14.3	13	2	Current study
Alpine Leaf-warbler *Phylloscopus affinis*	4.80	1.02	7.7	28.9	0.545	10.3	11	1	Current study
White-browed Tit-warbler *Leptopoecile sophiae*	5.80	0.92	7.8	35.5	0.334	9.9	3	1	Current study

#Data on Lifespan of species came from Tacutu et al. (2013) and Myhrvold et al. (2015).

References

Huang, J.; Liu, J.; Li, G.; Yan, H.; Li, S. Breeding Biology and Mating System of Brown Accentors *Prunella fulvescens* on the Tibet Plateau. *Bird Study* **2020**, *67*, 232-238.

Li, S.; Cheng, G.; Peng, W. Breeding Patterns of Asian Horned Larks (*Eremophila alpestris nigrifron*s) on the Tibet Plateau. *Wilson J. Ornithol*. **2016a**, *128*, 174-179.

Li, S.; Gao, H.; Liu, J.; Li, C.; Li, G.; Li, D. Life History Variation between Two Eurasian Tree Sparrow *Passer montanus* Populations at Different Altitudes. *Anim. Biol*. **2022**, *72*, 385-394.

Li, S.; Guo, C.; Zhang, G. Nesting Ecology of Tibetan Sand Martins *Riparia riparia* with Special Reference to Cooperative Breeding. *Ornithol*. *Sci*. **2016b**, *15*, 227-233.

Li, S.; Lu, X. Breeding Biology of Rock Sparrows *Petronia petroni*a in the Tibetan Plateau, with Special Reference to Life History Variation across Altitudes. *Acta Ornithol*. **2012a**, *47*, 19-25.

Li, S.; Lu, X. Reproductive Ecology of Isabelline Wheatears at the Extreme of Their Altitude Distribution. Ardeola **2012b**, 59, 301-307.

Li, S.; Peng, W.; Guo, C.; Lu, X. Breeding Biology of the Small Snowfinch Pyrgilauda davidiana on the Tibetan Plateau. *Forktail* **2013**, *29*, 155-157.

Li, S.; Peng, W.; Guo, C.; Lu, X. Factors Affecting Nest Success of the Oriental Skylark on the Tibetan Plateau. *Ornithol. Sci*. **2015a**, *47*, 19-25.

Li, S.; Qin, J.; Jin, Z.; Li, W.; Yan, H. An Experimental Test of the Concealment Hypothesis Using Oriental Skylark (*Alauda gulgula*) Nests on the Tibet Plateau. Russ. *J. Ecol.* **2018b**, *49*, 588-590.

Li, S.; Shi, R.; Li, W.; Li, G. Grazing Pressure Affects Offspring Sex Ratio in a Socially Monogamous Passerine on the Tibet Plateau. *J. Avian Biol.* **2018a**, *49*, e01660.

Li, Y.; Li, S.; Guo, C.; Zhang, G.; Zhou, Y.; Lu, X. Nest Helpers Improve Parental Survival but Not Offspring Production in a High-Elevation Passerine, the Ground Tit *Pseudopodoces humilis*. *Ibis* **2015b**, *157*, 567-574.

Liu, J.; Yan, H.; Li, G.; Li, S. Nest Concealment Is Associated with Reproductive Traits across Sympatric Bird Species. *Ecol. Evol*. **2021**, *11*, 14079-14087.

Myhrvold, N.P.; Baldridge, E.; Chan, B.; Sivam, D.; Freeman, D.L.; Ernest, S.K.M. An Amniote Life-History Database to Perform Comparative Analyses with Birds, Mammals, and Reptiles. *Ecology* **2015**, *96*, 3109.

Tacutu, R.; Craig, T.; Budovsky, A.; Wuttke, D.; Lehmann, G.; Taranukha, D.; et al. Human Ageing Genomic Resources: Integrated Databases and Tools for the Biology and Genetics of Ageing. *Nucleic Acids Res*. **2013**, *41*, 1027-1033.
